# Supplementary material for: Accelerated Skin Wound Healing Using Flexible Photovoltaic-Bioelectrode Electrical Stimulation
Source: Micromachines (Basel). 2022 Mar 31;13(4):561. doi: 10.3390/mi13040561 (PMC9032666; doi:10.3390/mi13040561)
Supplement: Supplementary file 1 [file micromachines-13-00561-s001.zip › micromachines-1636623-supplementary.pdf]

micromachines

Supplementary Materials

Photoelectric Properties

On a single cell level, the J–V characteristics of the devices were evaluated using the Newport Thermal Oriel 91159A solar simulator (for single cells) and the SAN-EI ELECTRIC XES-1004SE-200S solar simulator (for multi-cells) under AM 1.5G (100 mW cm^{-1}) (for modules). The length and breadth of each sub-cell were established by utilizing various evaporation masks for both single cells and modules, as well as for individual cells. Similarly, the active area of sub-cells was adjusted to be 1.25 cm^2 in size, depending on the flexible substrates used and the design pattern used in the experiment. The performance of single cells assessed with and without a 1.25 cm^2 mask turned out to be pretty comparable. The light intensity of a Newport Oriel 91150V-KG5 Si-based solar cell and a Newport Oriel PN 91150V Si-based solar cell were measured and calibrated, respectively. With the use of a Keithley 2400 source meter unit, the J–V characteristics were recorded. An Oriel Newport system (Model 74 125) equipped with a conventional Si diode was used to conduct tests in an open-air environment. Using a Newport 300 W lamp source, we were able to create monochromatic light. The solar cell simulator is used for testing and calibration. The spectral range simulates most of the solar spectral range at 300–1100 nm and is mostly overlaid on the absorption characteristic peaks of organic polymers. The power density is 100 mW/cm^2 and after the light source starts to warm up, it is necessary to set the voltage scanning range, scanning speed, waiting time, etc. Voc: open-circuit voltage; Jsc: short-circuit current density; FF: fill factor; PCE: photoelectric conversion efficiency.

Live subject statement

All animal studies were performed in compliance with the guidelines set by the Zhejiang and Guangdong Association for Laboratory Animal Science and are in compliance with the National Standard of the Peoples' Republic of China, called the Laboratory animal-Guideline for ethical review of animal welfare. The overall project protocols were approved by the Animal Ethics Committee of Ningbo University and Southern Medical University.

Incisional wound model

Eight-week-old C57BL/6 mice were randomized into four groups. Each mouse was anesthetized intraperitoneally with 1% sodium pentobarbital, and the skin was prepared. Then, the full-thickness wound including the panniculus carnosus muscle was made on the mid-back. After that, a 1 cm diameter punch biopsy instrument was placed with moderate force onto the dorsum of the mouse to create an impression of the circumference. Next, the middle of the outline region of skin was sharply excised along the outline with a pair of scissors. The excised tissue was full-thickness skin in depth, leaving the subcutaneous dorsal muscle exposed after excision. The percentage of wound contraction was calculated on the basis of the percentage of wounds reduced from the original wound size. The wound area was marked on a transparent tracing sheet and the wounds surface was measured with the help of the Image J program (National Institutes of Health, USA). The percentage of wound closure was measured on days 4, 7, and 10.

Histopathology Examination

The harvested skins were fixed in 4% phosphate-buffered paraformaldehyde for 12 h and embedded in paraffin. Ten micrometers of thick serial sections was cut from the paraffin-embedded blocks and underwent H&E and Masson staining. The photographs were

uploaded to an appropriate computer platform and were analyzed using the ImageJ analysis software from three randomly selected views of each specimen, respectively.

In vitro cell culture and electrical stimulation

To verify the molecular mechanism underlying the effects of ES in vitro, the murine fibroblast cell line NIH-3T3 was cultured in our modified cell line containing 10% FBS (fetal bovine serum), 100 U/mL penicillin, and 100 μg/mL streptomycin. Culture medium (DMEM) in high glucose. The passage when cells reached 80% confluence (see Figure 1). The culture environment can be in a medium containing 25 mM glucose (Sigma-Aldrich, MO, USA) and 0.2–0.4 mM BSA-conjugated palmitate (Sigma-Aldrich, MO, USA) for 24 h to simulate high glucose and high fat environments. One group received electrical stimulation with different electric field strengths, and the control group received no stimulation. Sterile metal platinum wires were placed in the culture medium during electrical stimulation, and the medium was changed every four hours.

Statistical analysis and interpretation

The data were reported as the mean minus the standard error of the mean (SE), and the GraphPad Prism 6.0 software was used to perform statistical analysis (GraphPad Software, CA, USA). The significance of the statistical difference between the stimulation groups and the control group was determined by applying the Student's *t*-test between the two groups. ANOVA was used to assess multiple group comparisons in this study. When a *p*-value was less than 0.05, it was deemed statistically significant.

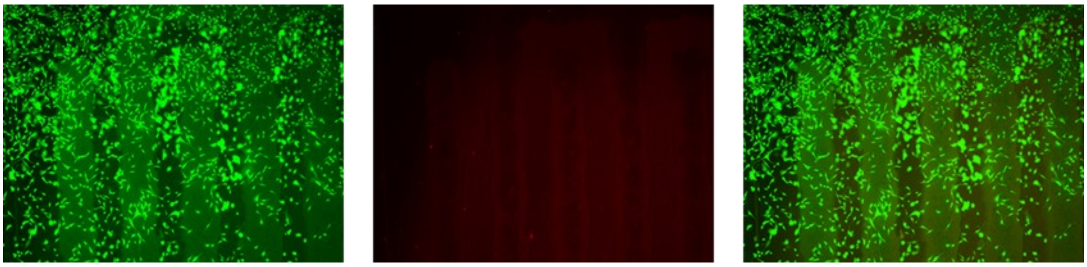

Figure S1. Live/dead staining images of fibroblasts cells 24 h treatment with sliver/PET substrate The scale bar is 100 μm.

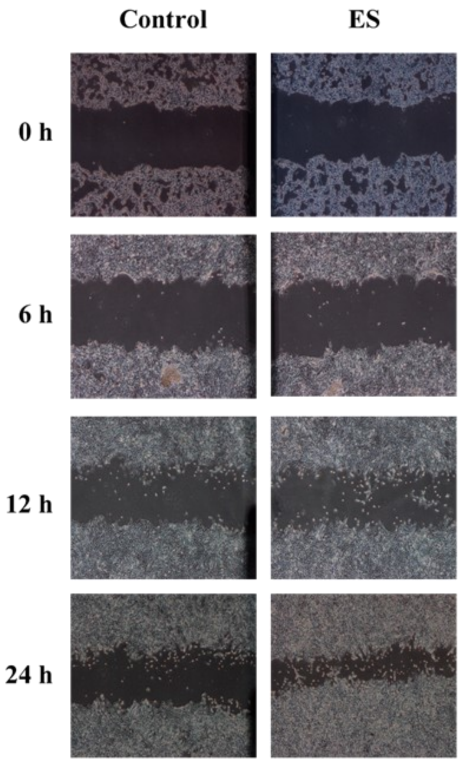

Figure S2. Images of transwell assays performed under electrical stimulation and quantitative analysis of migrating cells.

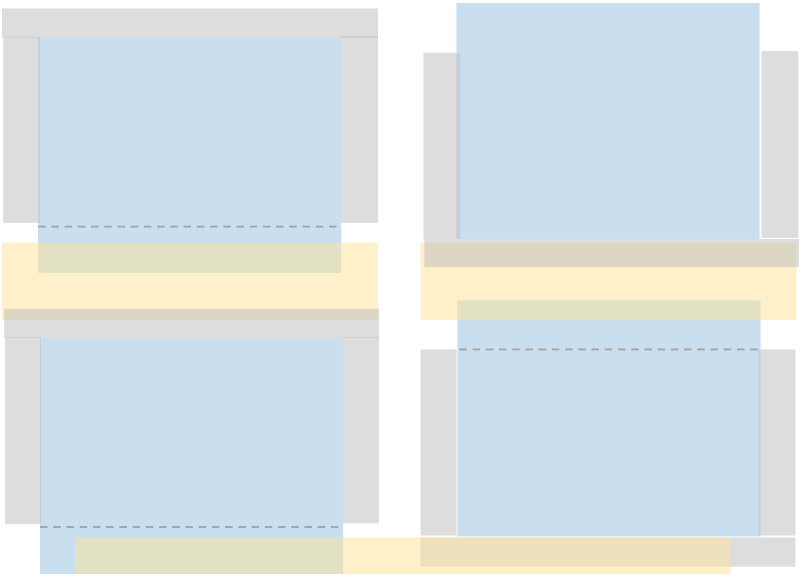

Figure S3. Single-junction large-area flexible organic solar cells connected in series through conductive silver paste.

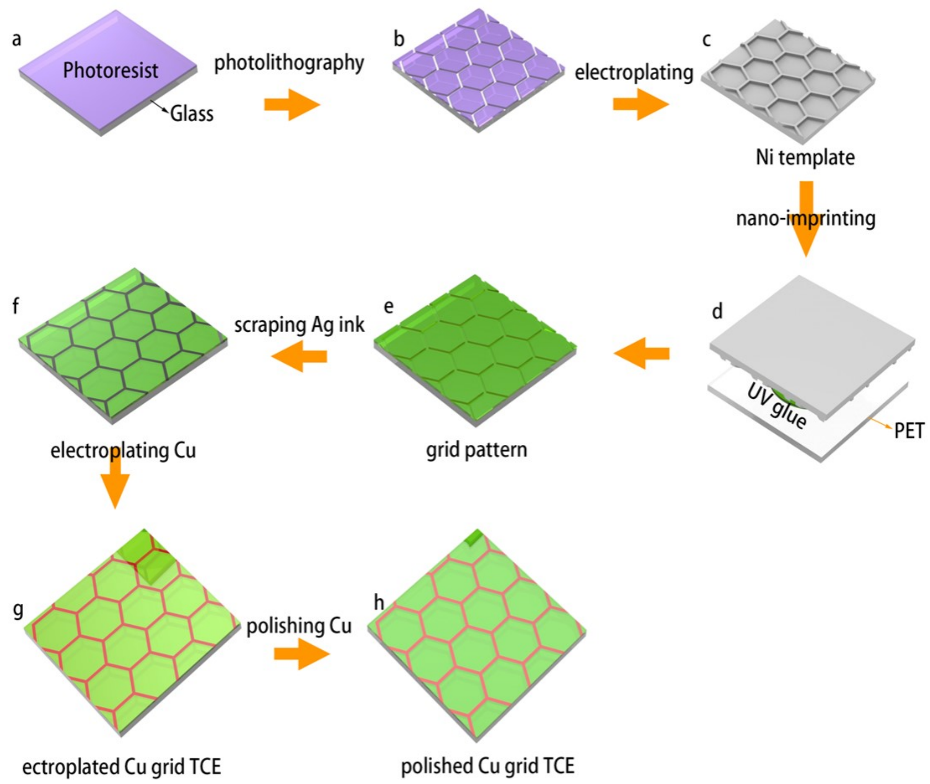

Hexagonal silver grid electrode fabrication process.

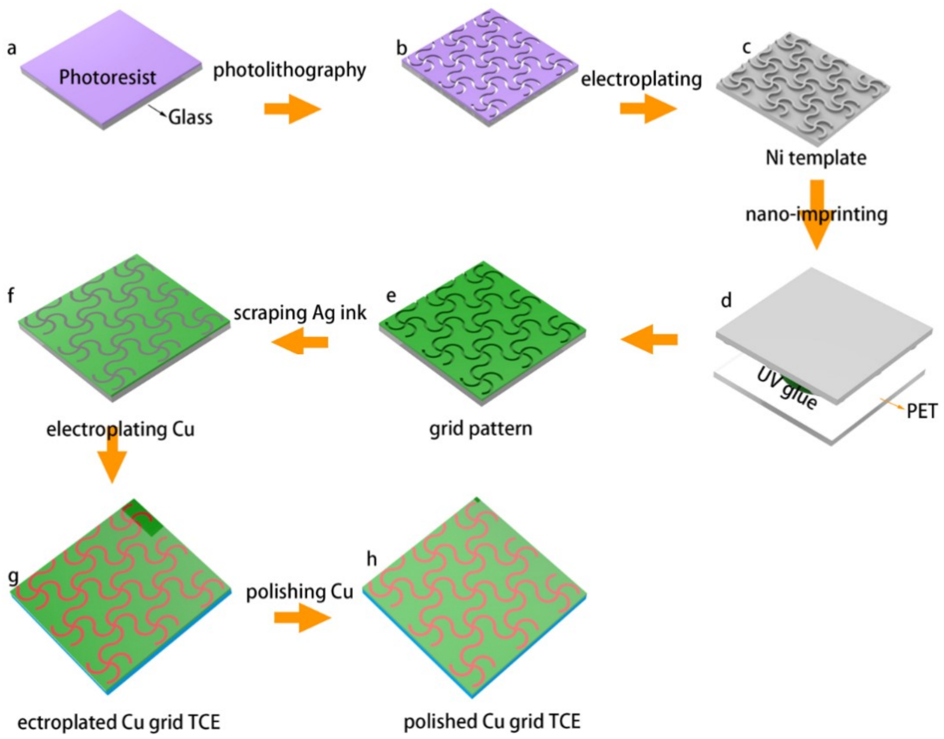

Serpentine silver grid electrode fabrication process.

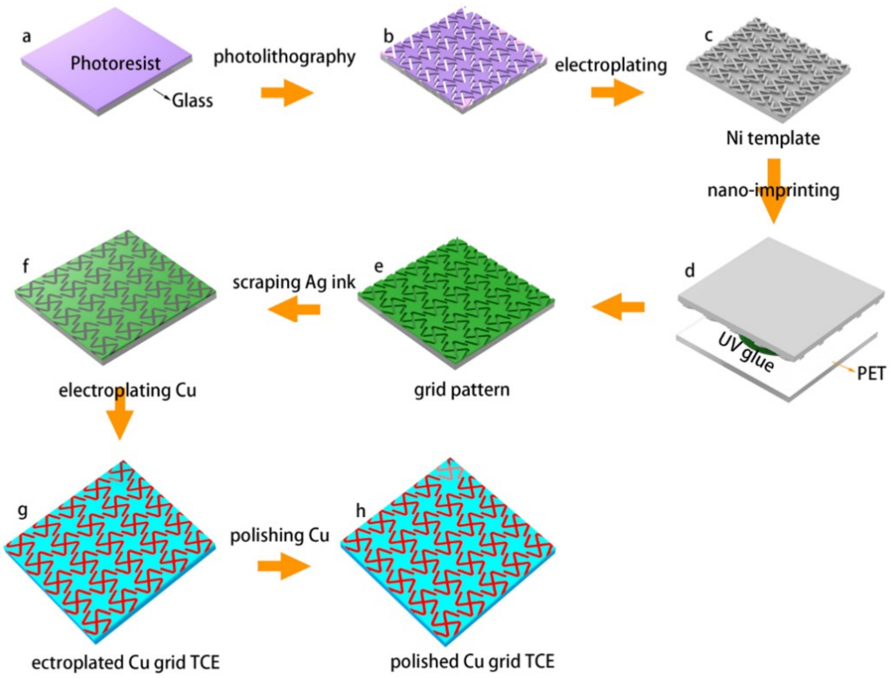

Crossed silver grid electrode fabrication process.

Figure S4. MEMS grid electrode manufacturing process diagram.

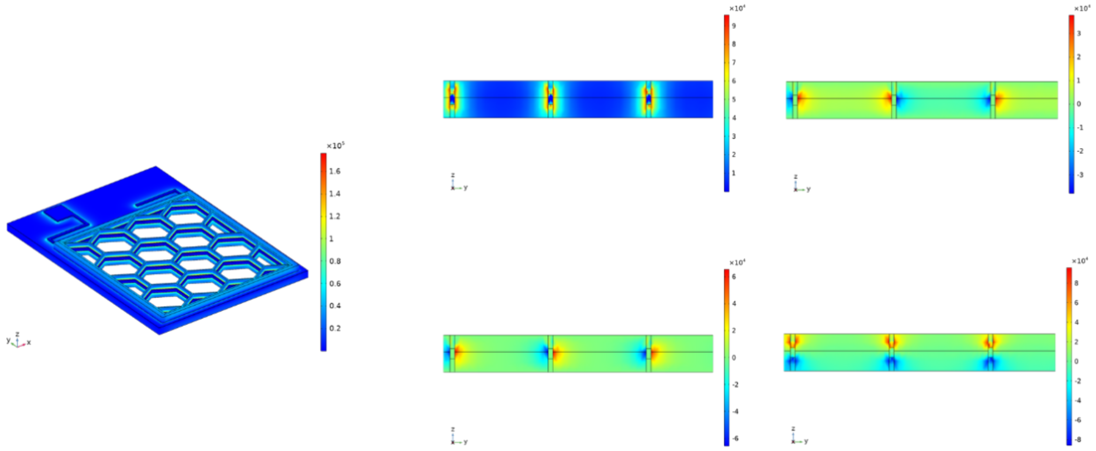

Hexagonal silver grid electrode: electric field modulus; x component; y component; z component.

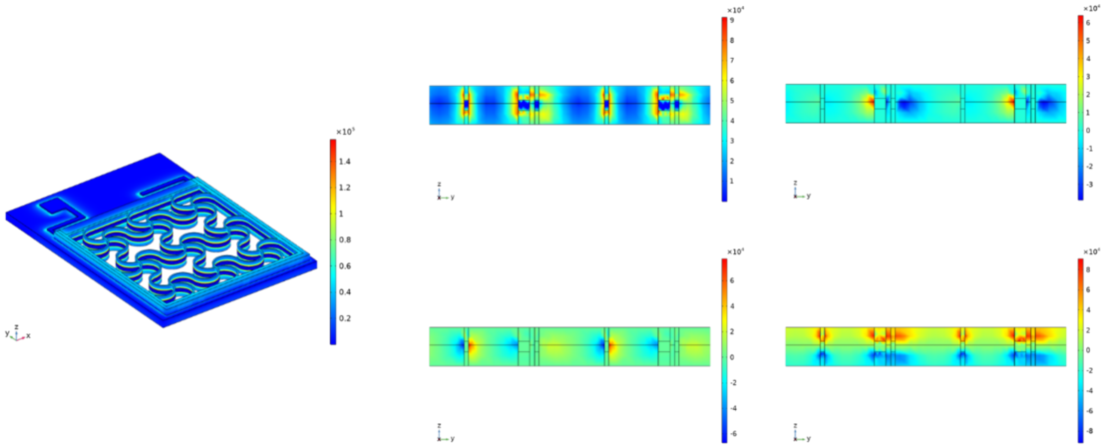

Serpentine silver grid electrode: electric field modulus; x component; y component; z component.

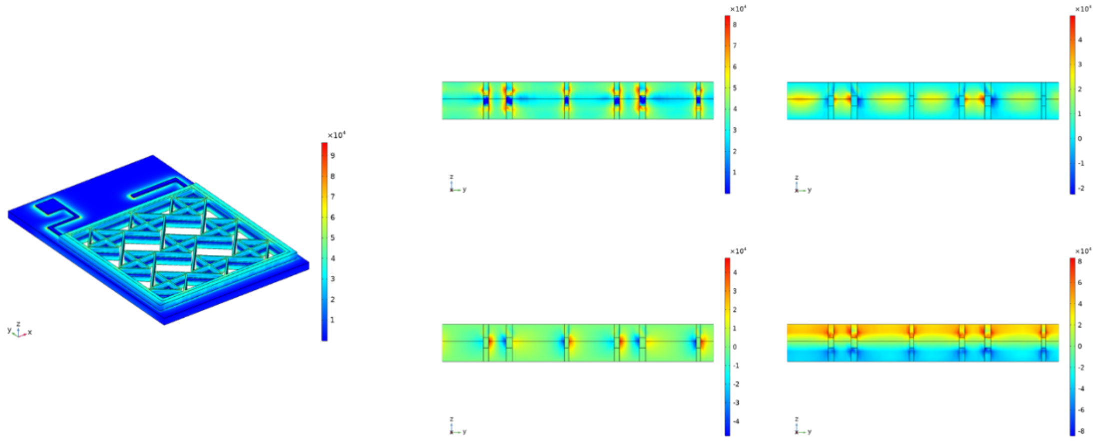

Crossed silver grid electrode: electric field modulus; x component; y component; z component.

Figure S5. Total electric field distribution and individual component vectors.

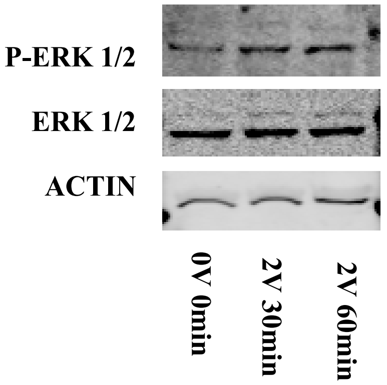

Figure S6. Electrical stimulation promoted proliferation and migration of fibroblasts in vitro. a) The protein levels of ACTIN, ERK1/2, and p-ERK1/2 in cells.

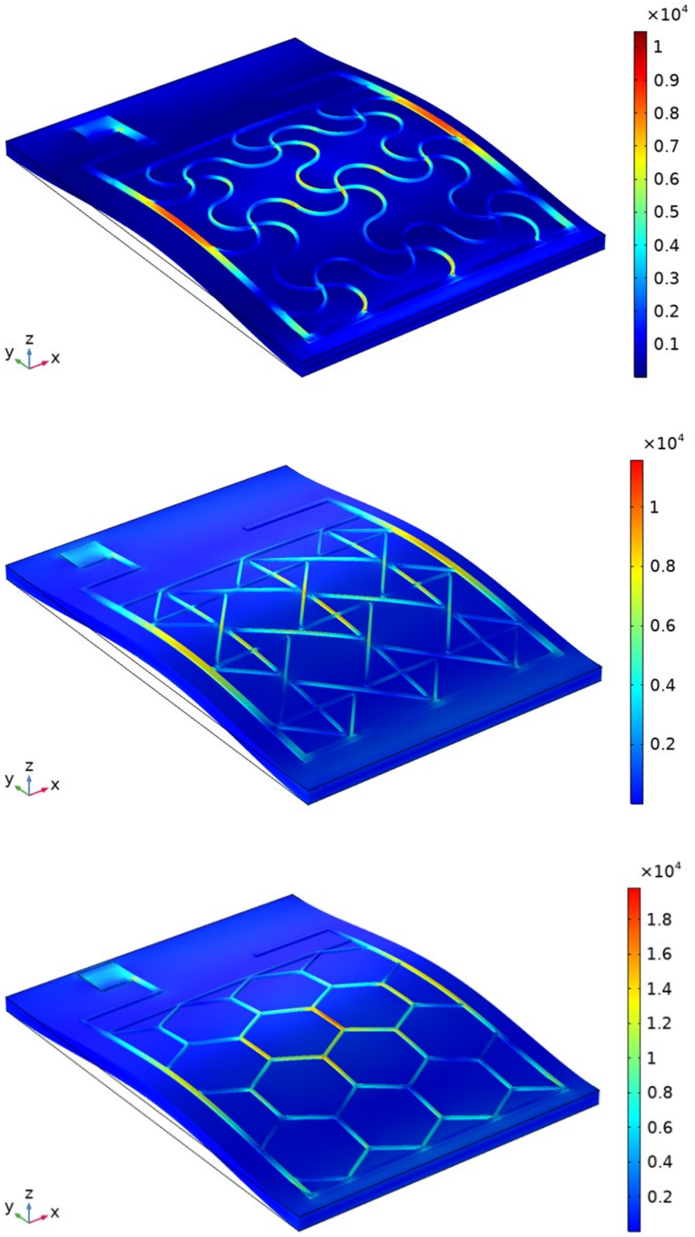

Figure S7. Electrode stress distribution.

Thanks for your suggestion. In fact, among different electrode structures, serpentine electrodes have certain mechanical advantages in addition to their advantages in electrical stimulation applications. We performed finite element simulation to obtain the stress distribution. It can be observed that, under the same conditions, the serpentine electrode has low stress, high load-bearing capacity, and strong tensile strength, which means that it is extremely suitable for the application environment, which fits the skin. For more details, please refer to the work published by us and our cooperative research groups. The density and Poisson's ratio of the substrate were 1100 kg m^{-3} and 0.48, respectively. The periodic serpentine structure is located at the half-thickness position. At an ambient temperature of 25 °C, the elastic modulus and Poisson's ratio of the metal are 104 GPa and 0.35, respectively. Experiments show that under different tensile strains, the serpentine electrode still maintains most of its conductive structures in a stable working state.
